# Abnormal T Cell Frequencies, Including Cytomegalovirus-Associated Expansions, Distinguish Seroconverted Subjects at Risk for Type 1 Diabetes

**DOI:** 10.3389/fimmu.2018.02332

**Published:** 2018-10-22

**Authors:** Robert Z. Harms, Kristina M. Lorenzo-Arteaga, Katie R. Ostlund, Victoria B. Smith, Lynette M. Smith, Peter Gottlieb, Nora Sarvetnick

**Affiliations:** ^1^Surgery-Transplant, University of Nebraska Medical Center, Omaha, NE, United States; ^2^Office of the Vice Chancellor of Research, University of Nebraska Medical Center, Omaha, NE, United States; ^3^Biostatistics, University of Nebraska Medical Center, Omaha, NE, United States; ^4^Barbara Davis Center for Diabetes, University of Colorado Anschutz Medical Campus, Aurora, CO, United States; ^5^Mary and Dick Holland Regenerative Medicine Program, University of Nebraska Medical Center, Omaha, NE, United States

**Keywords:** type 1 diabetes (T1D), mucosal associated invariant T cells (MAIT), CD4 T cells, CD8 T cells, TrialNet, autoimmunity, T regulatory cells (Treg), cytomegalovirus—HCMV

## Abstract

We analyzed T cell subsets from cryopreserved PBMC obtained from the TrialNet Pathway to Prevention archives. We compared subjects who had previously seroconverted for one or more autoantibodies with non-seroconverted, autoantibody negative individuals. We observed a reduced frequency of MAIT cells among seroconverted subjects. Seroconverted subjects also possessed decreased frequencies of CCR4-expressing CD4 T cells, including a regulatory-like subset. Interestingly, we found an elevation of CD57+, CD28–, CD127–, CD27– CD8 T cells (SLEC) among seroconverted subjects that was most pronounced among those that progressed to disease. The frequency of these SLEC was strongly correlated with CMV IgG abundance among seroconverted subjects, associated with IA-2 levels, and most elevated among CMV+ seroconverted subjects who progressed to disease. Combined, our data indicate discrete, yet profound T cell alterations are associated with islet autoimmunity among at-risk subjects.

## Introduction

Human type 1 diabetes (T1D) is characterized by the presence of antibodies targeting islet cell-associated antigens (i.e., autoantibodies) and the selective destruction of beta cells resulting in insulin deficiency (1). The destruction of beta cells is widely believed to be driven by autoreactive T cells (2). The evidence for this theory is generally indirect, comprising multiple reports of a variable T cell presence in and around islets [see for example (3)], including islet-antigen reactive CD8 T cells (4). Furthermore, islet-antigen reactive CD8 T cells have been isolated from the peripheral blood of type 1 diabetics and these can be induced to destroy beta cells in culture (5). *In vivo*, this unimpeded destruction by autoreactive CD8 T cells is considered to indicate an escape from tolerance, possibly due to dysfunctional regulatory T cells (Treg) among diabetics (6).

Defining the prodromal events which ultimately manifest in clinically-defined T1D remains a critical area of research. It is known that autoantibody presence often occurs several years prior to diagnosis (7), with functional aberrancy and reduction in the beta cell population preceding overt hyperglycemia (8, 9). While genetic factors can identify some risk, environmental variables are the ostensible drivers of T1D and its rising rate of incidence (10–12). In the absence of a well-characterized environmental trigger that may be avoided, early intervention following seroconversion is a worthy clinical goal for at-risk subjects (13). To that end, a thorough investigation of immune cells during the preclinical period is warranted. Such an investigation can reveal immune perturbations indicative of environmental exposures and/or regulatory dysfunction. These data can then be used to define biomarkers indicating risk, in the design of therapeutics, and in the unraveling of autoimmune mechanisms leading to beta cell decline.

Although a simply stated need, the identification of at-risk subjects with subsequent leukocyte isolation is a near Herculean labor. Importantly, an ongoing collaboration was established to meet this challenge. The T1D TrialNet Pathway to Prevention Study involves screening relatives of type 1 diabetics for autoantibodies to gauge risk, as well as a component for biosample acquisition (14). We utilized TrialNet PBMC samples to explore the hypothesis that peripheral T cell abundance is altered among seroconverted, at-risk subjects. We used flow cytometry to interrogate multiple T cell populations using a range of differentiation and lineage markers. Our results demonstrate profound changes in frequency of T cell subsets are associated with seroconversion, with several T cell lineages impacted including regulatory-like (Treg-like), cytotoxic (CD8), and mucosal associated invariant T (MAIT) cells. These data suggest substantial immunodeficiency underrides a likely pathogen response among seroconverted subjects.

## Materials and methods

### Patient samples

Cryopreserved PBMC were acquired as part of an ancillary study approved by TrialNet from the Pathway to Prevention study of 1st and 2nd degree relatives of individuals with type 1 diabetes. Aside from the familial association, study eligibility is limited to those 1st and 2nd degree relatives who are not on medication to control hyperglycemia, not currently using immunosuppressive or immunomodulatory agents, have no known severe active disease, and do not have diabetes or a history of diabetes. From this pool of subjects, we received 79 blinded samples comprising 3 subgroups of subjects. Twenty-five samples were from autoantibody negative (AA–) 1st and 2nd degree relatives of diagnosed type 1 diabetics or seroconverted, at-risk individuals. Having no autoantibodies, yet sharing some environmental and genetic risk, these samples constitute our control group. Importantly, these AA– subjects were not related to the seroconverted, at-risk individuals analyzed in our study. The remaining 54 subjects, labeled “seroconverted,” had previously seroconverted for one or more autoantibodies and are at increased risk of developing T1D (15, 16). Autoantibody testing was performed by TrialNet. Individuals are screened for GAD65, IA-2, and IAA. If any of these measurements were positive, they were also then screened for ZnT8 and ICA. Positivity thresholds were as follows: IAA, >0.010; GAD65, >0.032; ICA512, >0.049; GAD65H, >20; IA-2H, >5; ZnT8, >0.020; ICA, ≥10. Once an individual has tested positive for an autoantibody twice within one year, they are considered “autoantibody positive” or seroconverted to the production of autoantibodies targeting islet antigens. Among the seroconverted subset, 26 samples were from individuals who have not yet progressed to clinical disease, and for our analysis these are labeled “non-progressor.” The remaining 28 samples were from individuals who ultimately progressed to clinical disease. This subgroup is labeled as “progressor.” Our request for samples from TrialNet did not specify particular HLA-risk alleles, therefore our sample set constituted a mix of HLA types. Although we did request samples from subjects between 8 and 11 years old, upon unblinding the samples we discovered our subjects spanned a much broader age range. Patient data is presented in Table 1.

**Table 1:** Patient data.

**Status**		***n* (*n* female)**	**Variable**	**Mean**	**SD**	**Median**	**Min**	**Max**
AA-		25 (11)	Age	12.2	2.7	12.0	7.7	16.9
			BMI	19.7	5.4	18.7	14.5	35.6
			HbA1c	5.1	0.3	5.1	4.5	5.6
Seroconverted		54 (23)	Age	10.9	4.6	10.3	4.7	26.8
			BMI	20.4	5.5	19.1	14.0	45.7
			HbA1c	5.1	0.3	5.1	4.3	6.0
Seroconverted	Non-progressor	26 (10)	Age	10.6	4.0	9.9	4.8	21.8
			BMI	19.8	4.6	18.2	14.0	29.8
			HbA1c	5.0	0.3	4.9	4.5	5.8
	Progressor	28 (13)	Age	11.3	5.1	10.8	4.7	26.8
			BMI	21.1	6.2	20.7	14.8	45.7
			HbA1c	5.2	0.4	5.3	4.3	6.0

### Flow cytometry

Surface panel design and analysis was performed using a BD LSR II equipped with 5 lasers: UV (355 nm) for Brilliant Ultraviolet (BUV) 395 and 737 and LiveDead UV Blue; Violet (405 nm) for Brilliant Violet (BV) 421, 510, 605, 650, 711, and 785; Blue (488 nm) for FITC and PerCP-Cy5.5; Yellow-Green (561 nm) for PE, PE-CF594, and PE-Cy7; and Red(633 nm) for APC and Alexa Fluor(AF) 700. The following antibodies and reagents were used: from Biolegend CCR7(PE, clone G043H7), CCR4(PE-Cy7, clone L291H4), CD14(BV711, clone M5E2), CD19(BV711, clone HIB19), CD27(BV785, clone O323), CD57(FITC, clone HCD57), CD45RA(PerCP-Cy5.5, clone HI100), Vα7.2(BV605, clone 3C10), CD45(AF700, clone HI30), Streptavidin-APC; from BD Biosciences CXCR5(BV421, clone RF8B2), CD3(BUV737, clone UCHT1), CD4(BUV395, clone RPA-T4), CD8(BV510, clone RPA-T8), CD28(PE-CF594, clone CD28.2), CD161(BV650, clone DX12); from eBioscience CD127(Biotin, clone eBioRDR5); from life technologies LiveDead UV Blue. Cryopreserved PBMC were thawed and rested overnight in X-VIVO 15 (Lonza) supplemented with human AB serum (MP Biomedicals) at 2% v/v at 37° C with 5% CO_2_. Samples were then counted and distributed to 96 well plates for labeling following methods described previously (17) with two modifications: Fc receptors were blocked using human IgG (Sigma Aldrich) and antibody cocktails were prepared using Brilliant Stain Buffer (BD Biosciences).

For FOXP3 analysis, lymphocytes from healthy human donors were acquired from the UNMC elutriation core facility, cryopreserved, thawed, rested and surface labeled as described above. We then utilized the True-Nuclear Transcription Factor Buffer set (BioLegend) according to manufacturer's recommendations. In panel validation, we compared PE-conjugated FOXP3 clones 206D (BioLegend), PCH101 (eBioscience), and 259D/C7 (BD Biosciences) and clone 206D gave the greatest signal-to-noise ratio (data not shown). The final panel included the aforementioned FOXP3, CD25 (VioBright FITC, clone 4E3, Miltenyi), and the conjugated-clones and reagents listed above for LiveDead, CD3, CD4, CXCR5, CCR4, and CD127 with streptavidin-APC.

Data analysis was conducted in a blinded fashion using FlowJo (v10.2, TreeStar). All samples were pregated to exclude doublets, debris, dead cells, and non T cells (lineage negative) and then multiple subsets were identified and gated according to known T cell differentiation pathways. A representative gating procedure for all subsets explored is shown in Supplemental Figure 1. Following analysis, raw data files and tabular results were uploaded to TrialNet, upon which we received sample identification, clinical data, and autoantibody levels in order to perform statistical analysis.

### Measurement of anti-viral immunoglobulins

We received blinded vials of plasma from the majority of the subjects in our PBMC analysis: 25/25 AA–, 23/26 non-progressors, and 27/28 progressors. Prior to performing assays, the vials of plasma were thawed, divided into several aliquots, refrozen, and stored at −80°C in order to run several assays and limit sample degradation from multiple freeze/thaw cycles. The following anti-viral immunoglobulin ELISAs from IBL America were performed according to manufacturer's directions: CMV IgM (IB19206), CMV IgG (EG101), EBV VCA IgM (IB79231), EBV VCA IgG (IB79230), enterovirus IgM(IB05053), and enterovirus IgG(IB05052). All sample dilutions contained Heteroblock (Omega Biologicals Inc.) to eliminate potential interference resulting from the presence of heterophilic antibodies. Optical densities were measured using a Synergy H1 Hybrid Multi-Mode Reader (BioTek Instruments, Inc.). Sample positivity for viral immunoglobulins was determined following calculations defined per assay. If sample positivity was equivocal, the sample was retested. If the sample was also equivocal on the follow-up test, it was labeled as “gray” for purposes of stratification and treated as negative. Upon completion of our plasma analysis, tabular data was uploaded to TrialNet. We then received sample identification data for pairing with our PBMC results and subsequent statistical analysis.

### Statistics

For mean comparisons, natural log transformation was applied to the data prior to analysis. For comparison of two groups, we used Student's *T*-test and for comparisons of three groups, we used analysis of variance (ANOVA). If the results of the ANOVA were significant, then pairwise comparisons were made, adjusting for multiple comparisons with Tukey's method. For correlations, data were not transformed and we used Spearman's Rank-Order correlation. In all cases, figures and descriptive data depict non-transformed data to facilitate interpretation. Figures were made using Prism (v6.03 GraphPad Software Inc.), and data was analyzed using Prism and SAS software versions 9.4 (SAS Institute Inc., Cary, NC).

## Results

### Reduced frequencies of mucosal associated invariant T cells (MAIT) in seroconverted subjects

MAIT cells are regularly found in human blood, the liver, and the gastrointestinal mucosa (18), and respond to microbially-derived vitamin B metabolites presented through MR1 (19). MAIT cells are capable of directly lysing bacterially-sensitized and infected target cells (20, 21) and can produce the proinflammatory cytokines IFN-γ, TNF-α, and IL-17 (18). Phenotypically, in the absence of MR1-loaded multimers, MAIT cells are unambiguously identifiable in blood by the high CD161 expression, presence of Vα7.2 T cell receptor, and T cell lineage marker CD3 (18, 22). The circulating MAIT cell lineage in healthy subjects is also regularly CD28+, CD127+, CD45RA^low^, and CCR7^low^ (18, 23), but exceptions to this phenotype do exist in certain disease states [e.g., chronic HIV (24)], as well as in fetal tissue and cord blood (25, 26). MAIT cells are generally CD8+ (~85%) with the remaining being chiefly DN (CD4–, CD8–), although CD4+ and DP (CD4+, CD8+) groups are frequently observable in scant amounts in healthy donors. The functional differences among these MAIT cell subsets have not been fully clarified, although it has recently been reported that the CD8+ subset possesses somewhat greater effector potential than the DN subset (27).

**Figure 1:**
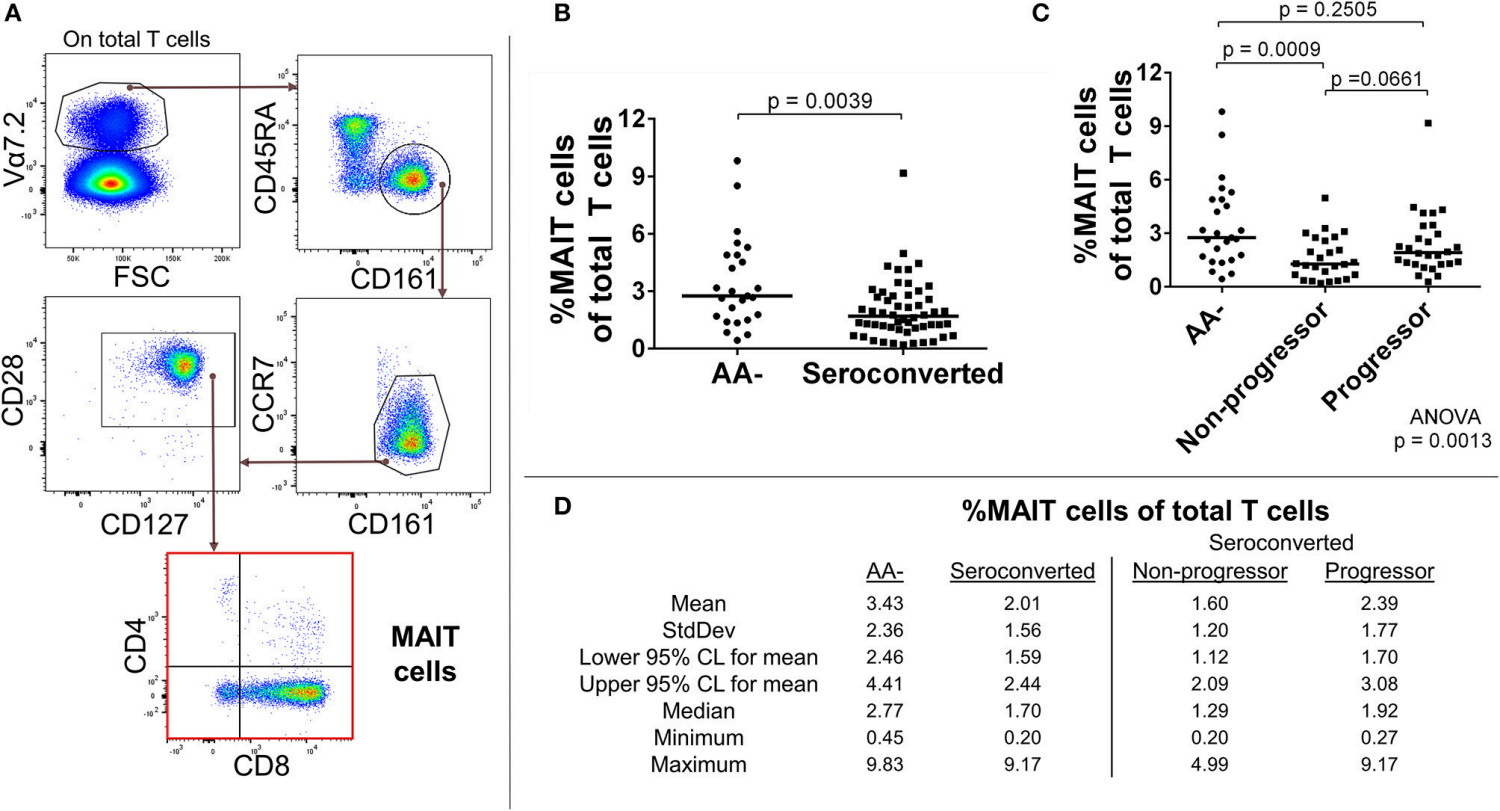
Seroconverted subjects have reduced frequencies of MAIT cells. **(A)** Starting from total T cells, MAIT cells were identified as Vα7.2+, CD45RA-, CD161+, CCR7^dim/−^, CD28+, CD127+ events. **(B)** Seroconverted subjects have reduced frequencies of MAIT cells in comparison to AA– subjects. Reduced frequencies were most acute in the CD8+, CD4– (CD8+), and CD8–, CD4– (DN) compartments (Supplemental Figure 2). **(C)** Upon dividing seroconverted subjects according to disease progression, we observed that reduced frequencies of MAIT cells were most prominent among non-progressors. **(D)** Descriptive statistics for the frequency of MAIT cells of total T cells for all groups compared. For figures **(B,C)**, bars represent median. Statistical tests fully described in section Materials and Methods.

We identified total MAIT cells as CD3+, Vα7.2+, CD161+, CD45RA^low^, CCR7^low^, CD28+, CD127+ events and also divided them by CD4 and CD8 expression (Figure 1A). With this approach, we observed a sharply reduced frequency of MAIT cells among seroconverted individuals (Figures 1B,D). This reduction extended into all MAIT cell subsets as defined by CD4 and CD8 expression, and appeared most acute within the major compartments identified as CD8+ and DN (Supplemental Figures 2A,B). When evaluating the seroconverted subjects according to disease progression, we found the non-progressors harbored the fewest MAIT cells, while the reduction among the progressors was not significant (Figures 1C,D). These data indicate that a MAIT cell reduction is associated with seroconversion and this effect is slightly ameliorated among those who progress to disease.

### Short-lived effector-like CD8 T cells are expanded in seroconverted individuals and this is most pronounced among those who progressed to disease

Conventional αβ CD8 T cells are the chief cytotoxic arm of adaptive immunity. Upon licensing and activation, CD8 T cells rapidly proliferate in order to destroy microbially-infected or transformed cells (28). Following the eradication of targets, effector cells die off while memory populations remain in tissue and circulation. This memory provides a quick recall response to repeat offenders and is considered a hallmark of T cell immunity. Outside of this role as defenders, CD8 T cells have been incriminated in the pathogenesis of several autoimmune diseases (29).

**Figure 2:**
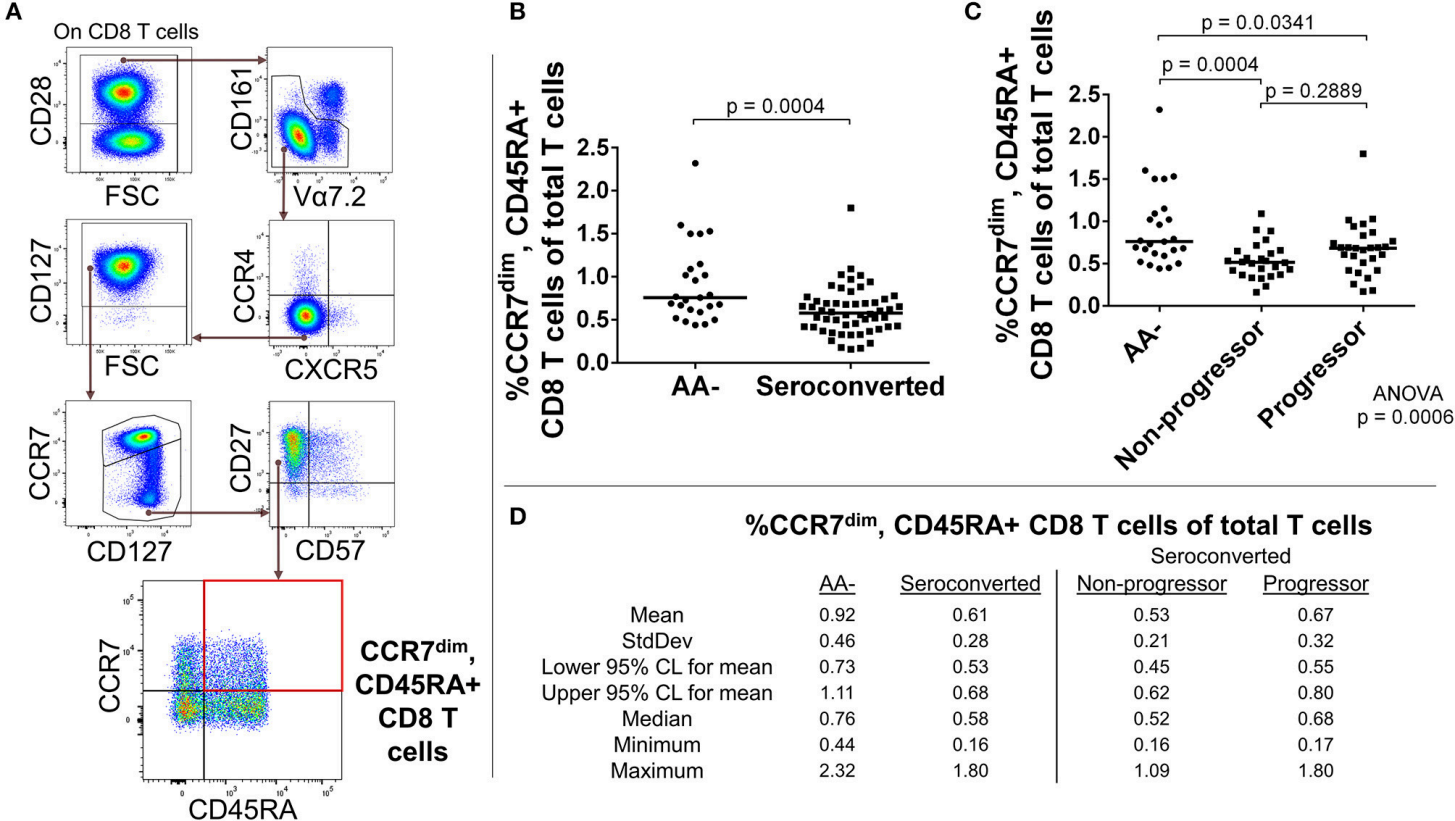
Seroconverted subjects have reduced frequencies of CD45RA+, CCR7^dim^ memory-like CD8 T cells. **(A)** Starting from total CD8 T cells. CD45RA+, CCR7^dim^ memory-like CD8 T cells were identified as CD28+, MAIT–, CCR4–, CXCR5–, ${\text{CD}127}_{,}^{+/\text{high}}$ CCR7^dim^, CD27+, CD57–, and CD45RA+. **(B)** Seroconverted subjects have reduced frequencies of CD45RA+, CCR7^dim^ memory-like CD8 T cells in comparison to AA- subjects. Reduced frequencies of CD45RA+, CCR7– memory-like CD8 T cells were also observed (Supplemental Figure 3). **(C)** Upon dividing seroconverted subjects according to disease progression, we observed that reduced frequencies of CD45RA+, CCR7^dim^ memory-like CD8 T cells were most prominent among non-progressors. **(D)** Descriptive statistics for the frequency of CD45RA+, CCR7^dim^ memory-like CD8 T cells of total T cells for all groups compared. For figures **(B,C)**, bars represent median. Statistical tests fully described in section Materials and Methods.

In our analysis of CD8 T cells, we first divided CD8 T cells according to presence or absence of costimulatory protein CD28. Among the CD28+ CD8 T cells, we removed MAIT cells and analyzed the remaining CD8 T cells for expression levels of CCR4, CXCR5, CD45RA, CD27, CD127, CCR7, and CD57. Similar to what was observed among MAIT cells, seroconverted subjects possessed reduced proportions of CD45RA+, CCR7^dim/−^, CD57–, CD27+, CD127^high^, CXCR5–, CCR4– CD8 T cells compared to AA– subjects (Figures 2A,B,D and Supplemental Figure 3). This combination of markers can best be described as a CD45RA+ memory subset following current differentiation paradigms (30, 31). Upon dividing the seroconverted into non-progressor and progressor subsets, we found that the non-progressors were significantly reduced in proportion, while the progressors did not approach significance (Figures 2C,D and Supplemental Figure 3).

**Figure 3:**
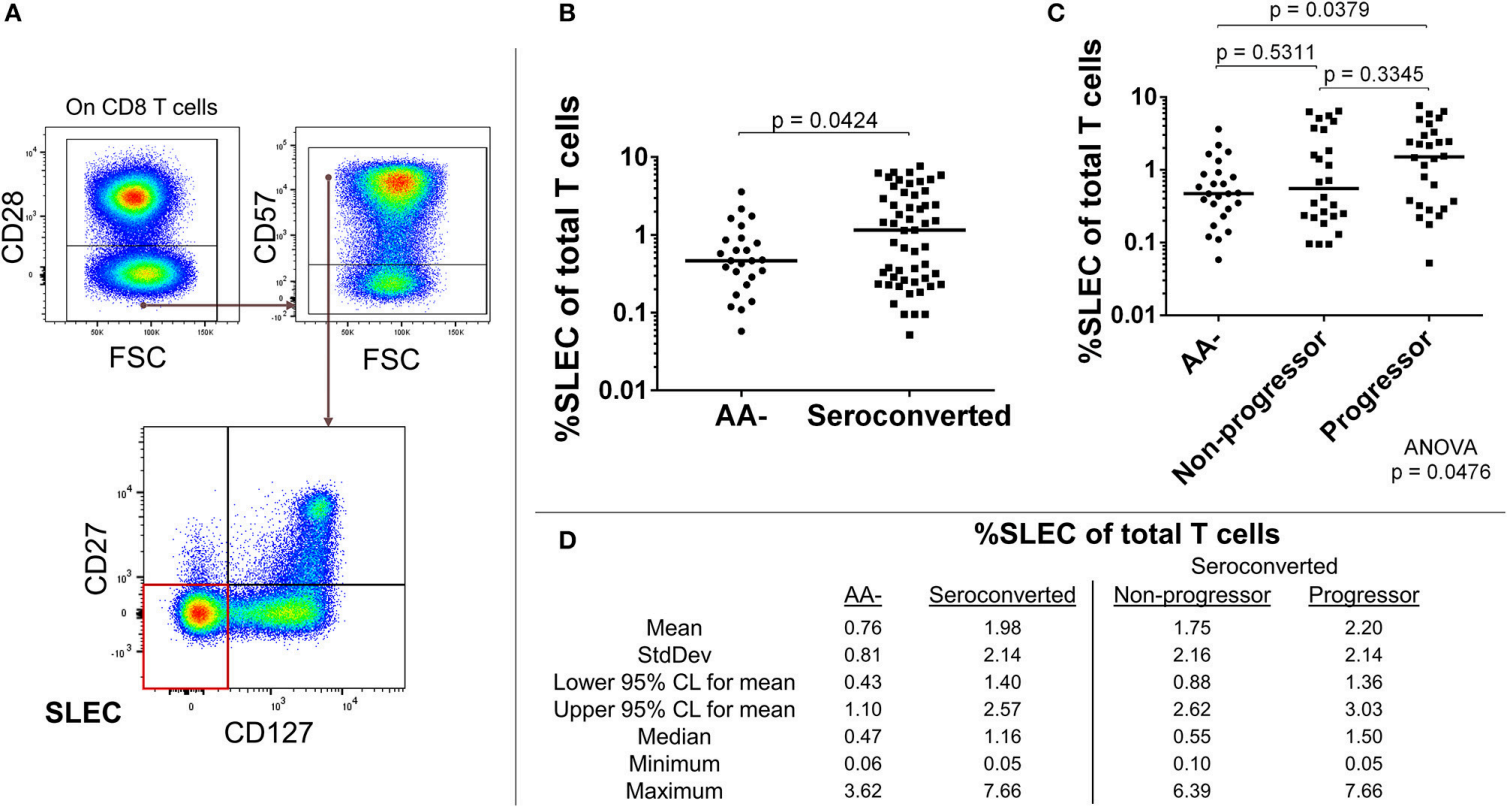
Seroconverted subjects have elevated frequencies of short-lived effector-like cells (SLEC) and this expansion was most prominent among those that progressed to disease. **(A)** Starting from total CD8 T cells, SLEC were identified as CD28–, CD57+, CD27–, and CD127–. **(B)** Seroconverted subjects have increased frequencies of SLEC in comparison to AA– subjects. **(C)** Upon dividing seroconverted subjects according to disease progression, we observed that elevated frequencies of SLEC were most prominent among progressors. **(D)** Descriptive statistics for frequency of SLEC of total T cells for all groups compared. For figures **(B,C)**, bars represent median. Statistical tests fully described in section Materials and Methods.

The absence of CD28 on human T cells is indicative of chronic stimulation (32). We divided CD28- CD8 T cells by expression of CD57, CD127, and CD27 to characterize this antigen-experienced compartment. Our analysis revealed an elevated frequency of CD127–, CD27–, CD57+, CD28– CD8 T cells among seroconverted subjects (Figures 3A,B,D). This combined phenotype indicates terminal differentiation, cytotoxic potential via perforin production, and short-lived status (33–36). Thus, we abbreviated their phenotype as SLEC for short-lived effector-like cells. Intriguingly, this expansion of SLEC was most prominent among progressors (Figures 3C,D), suggesting an acute pathogen response may be associated with disease progression.

### CCR4-expressing CD4 T cell subsets, including CD127^dim^ Treg-like cells, are reduced in seroconverted subjects

C-C chemokine receptor 4 (CCR4) expression among CD4 T cells suggests previous T cell receptor engagement (37, 38) as well as chemotactic responsiveness to thymus and activation-regulated chemokine (TARC), macrophage-derived chemokine (MDC), and chemokine like factor 1 (CKLF1) (39–41). Rather than solely indicating “Th2” status, CCR4– expressing CD4 T cells can be enriched for IFN-γ, IL-22, IL-17, and/or IL-4 production, as well as possess regulatory function (42–46).

**Figure 4:**
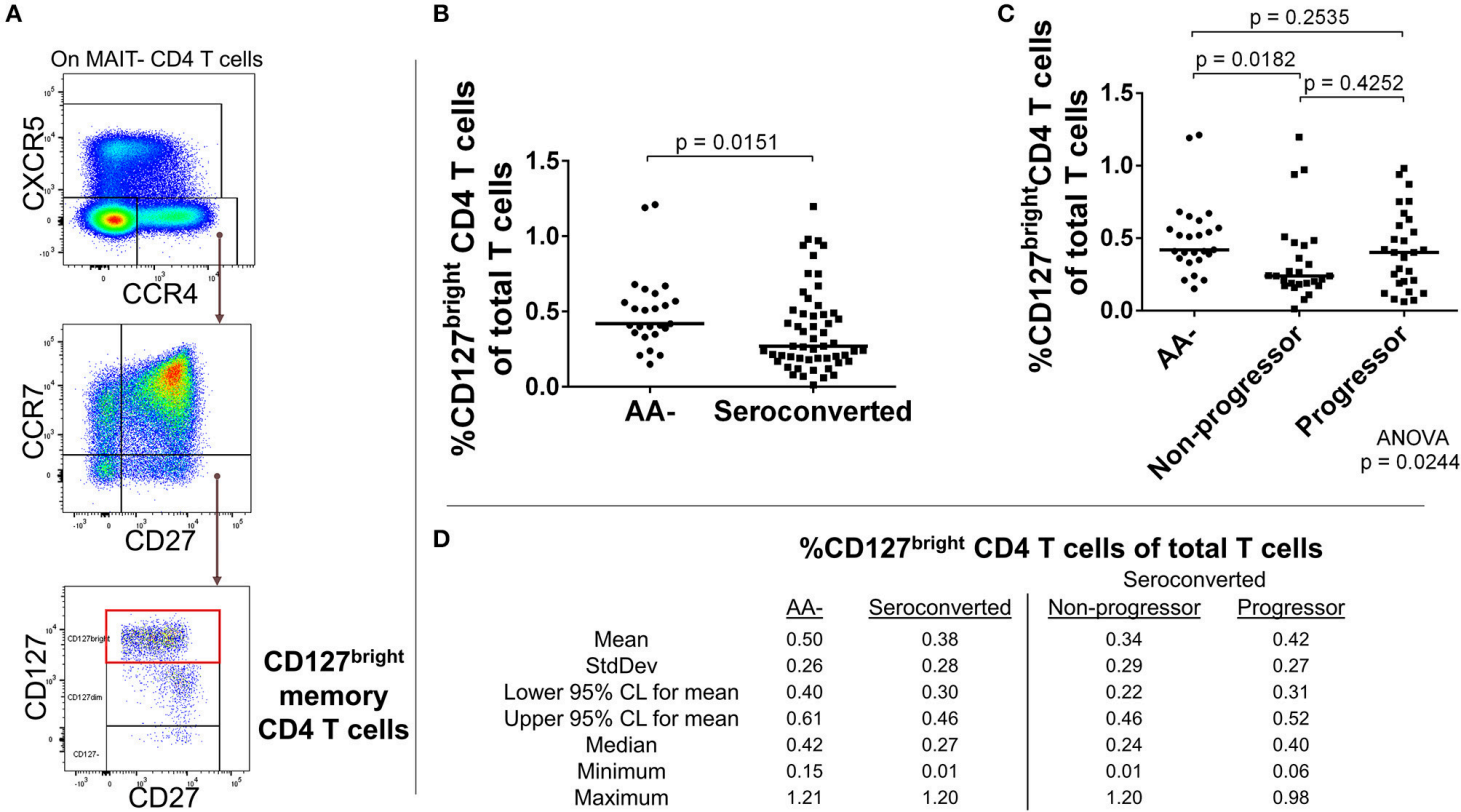
Seroconverted subjects have reduced frequencies of CCR4+, CD127^bright^ memory CD4 T cells. **(A)** Starting from total MAIT– CD4 T cells, CD127^bright^ memory T cells were identified as CCR4+, CXCR5–, CCR7–, CD27+, CD127^bright^ events. **(B)** Seroconverted subjects have reduced frequencies of CD127^bright^ memory CD4 T cells in comparison to AA– subjects. CCR4-expressing terminally differentiated and T follicular helper-like CD4 T cells were also reduced in frequency (Supplemental Figure 4). **(C)** Upon dividing seroconverted subjects according to disease progression, we observed that reduced frequencies of CD127^bright^ memory CD4 T cells were most prominent among non-progressors. **(D)** Descriptive statistics for the frequency of CD127^bright^ memory CD4 T cells of total T cells for all groups compared. For figures **(B,C)**, bars represent median. Statistical tests fully described in section Materials and Methods.

**Figure 5:**
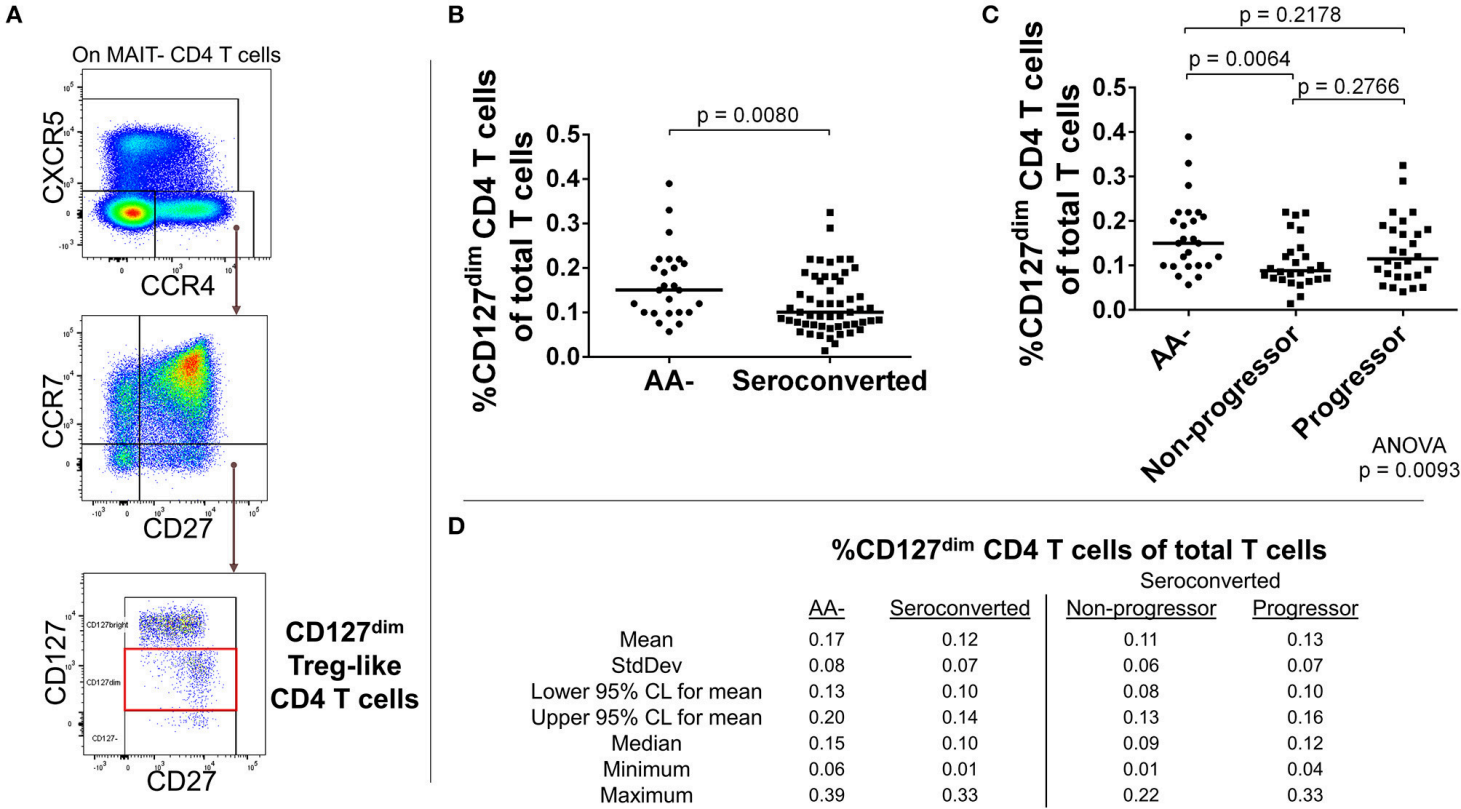
Seroconverted subjects have reduced frequencies of CCR4+, CD127^dim^ Treg-like CD4 T cells. **(A)** Starting from total MAIT– CD4 T cells, CD127^dim^ Treg-like cells were identified as CCR4+, CXCR5–, CCR7–, CD27+, CD127^dim^ events. **(B)** Seroconverted subjects have reduced frequencies of CD127^dim^ Treg–like cells in comparison to AA– subjects. **(C)** Upon dividing seroconverted subjects according to disease progression, we observed that reduced frequencies of CD127^dim^ Treg-like CD4 T cells were most prominent among non-progressors. **(D)** Descriptive statistics for the frequency of CD127^dim^Treg-like CD4 T cells of total T cells for all groups compared. For figures **(B,C)**, bars represent median. Statistical tests fully described in materials and methods.

Our analysis of CD4 T cells from TrialNet donors revealed reductions in frequency of three CCR4– expressing subsets among seroconverted subjects. Memory (CD127^bright^, CD27+, CCR7–, CCR4+, CXCR5–; Figures 4A–D), Treg-like (CD127^dim^, CD27+, CCR7–, CCR4+, CXCR5–; Figures 5A–D), and T follicular helper-like (CCR4+, CXCR5+, CD161–; Supplemental Figure 4) CD4 T cell subsets were all reduced among seroconverted subjects. For each compartment, the reduction in frequency was most profound among non-progressors and approached “normal” levels among progressors.

**Figure 6:**
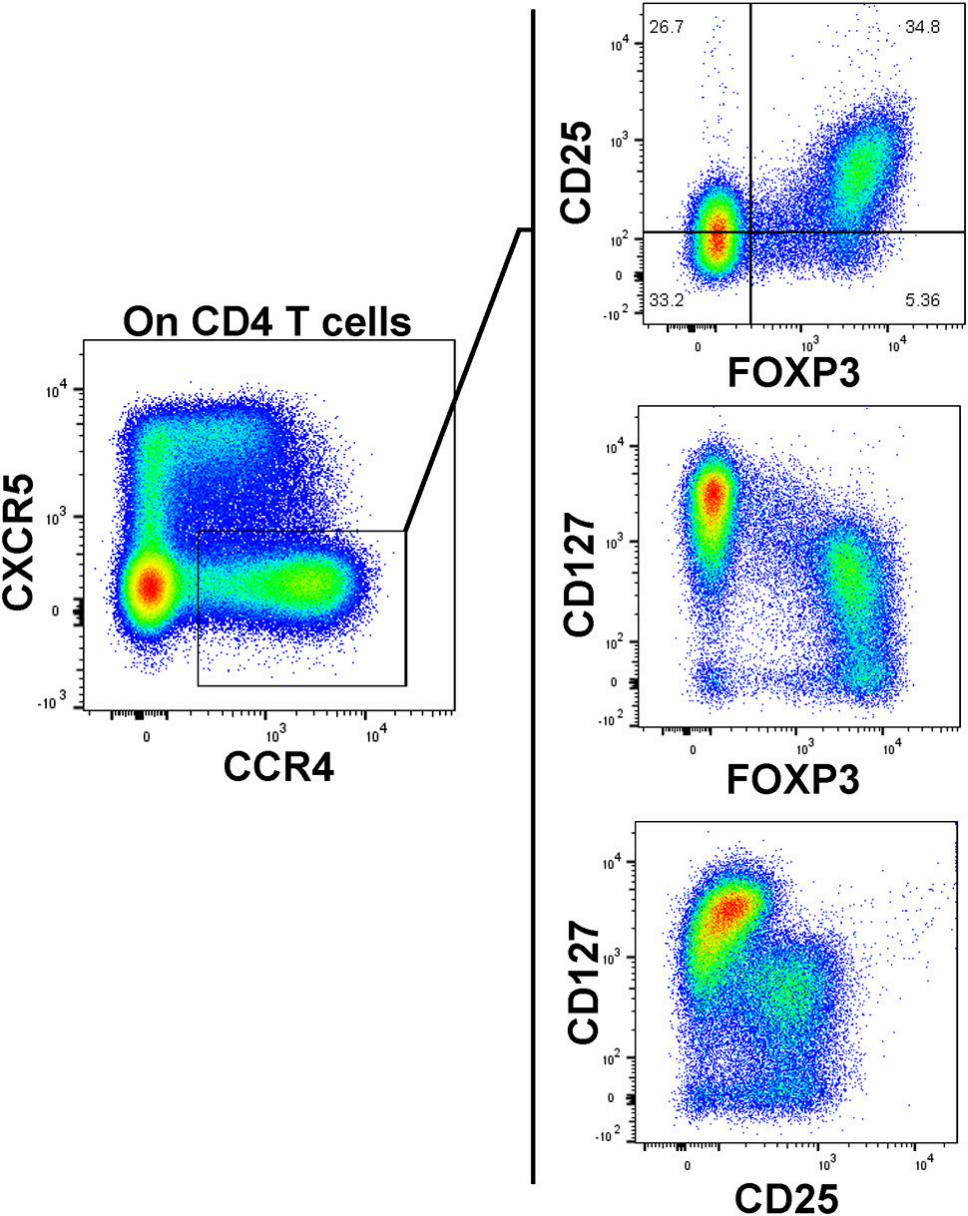
CCR4+, CXCR5– CD127^dim^ CD4 T cells possess a regulatory phenotype. A substantial population of FOXP3+, CD25^high^ events resides in the CXCR5–, CCR4+ subset. These FOXP3+ events generally exhibited low CD127 expression. Data is representative of 3 healthy subjects analyzed.

As our T cell panel did not include CD25 nor FOXP3, we were unable to cross-confirm regulatory phenotype among the Treg-like, CCR4-expressing CD4 T cells that we observed contracted among TrialNet samples. However, we analyzed CD4 T cells from 3 healthy human donors and found that the CCR4+, CXCR5– compartment possesses a substantial FOXP3+, CD25+ subset, which is also CD127^dim/−^, a combined phenotype indicating regulatory potential (Figure 6). This is in agreement with previous studies indicating CCR4+ and CD127^dim/low^ CD4 T cells contain Tregs (47–49). Thus, it's highly likely that the observed Treg-like CCR4+ CD4 T cells we found to be reduced among seroconverted subjects contains T cells with regulatory potential.

### Frequencies of CD127^dim^ Treg-like cells are positively correlated with CD127^bright^ memory CD4 T cells, while CD45RA+, CCR7^dim^ memory CD8 T cells and SLEC are positively correlated with islet autoimmunity

We observed altered frequencies of MAIT, CCR7^dim/−^ CD45RA+ CD8 memory, SLEC, and CCR4+ CD4 T cells, including memory and Treg-like subsets. As mentioned previously, it's widely presumed that T1D is driven by autoreactive CD8 T cells which have escaped tolerance. Since Tregs can effectively suppress T cell proliferation *in vitro* (50), it's plausible that such subsets may impact T cell expansions and contractions *in vivo*. Therefore, we examined some of the CD4 and CD8 T cell subsets described above for relationships with our CD127^dim^ Treg-like subset.

**Figure 7:**
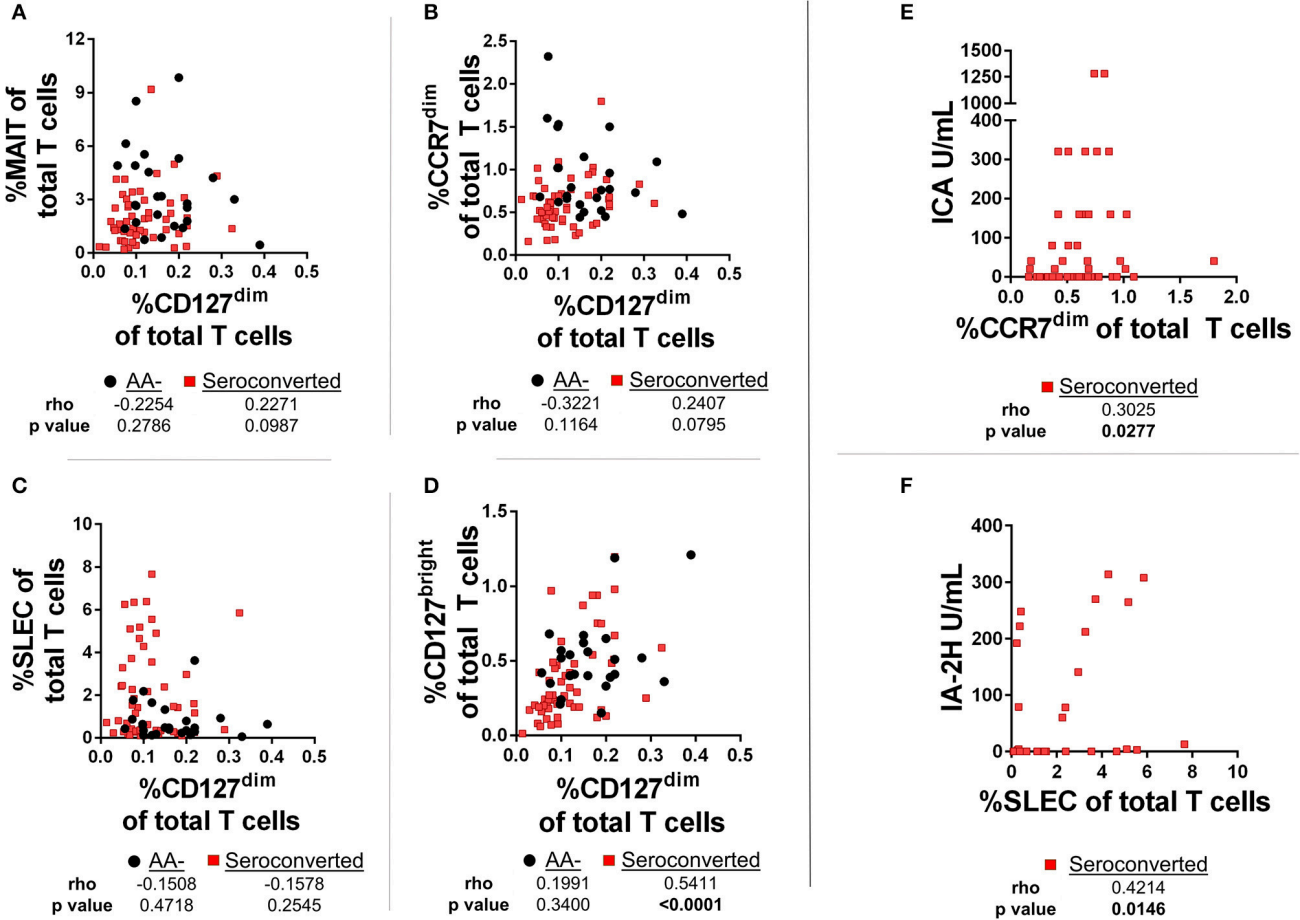
Frequencies of CD127^dim^ Treg-like cells are positively correlated with CD127^bright^ memory CD4 T cells among seroconverted subjects. **(A)** We observed no significant correlation among frequencies of CD127^dim^ Treg-like cells and MAIT cells. **(B)** Although not correlated among AA- subjects, frequencies of CD127^bright^ memory and CD127^dim^ Treg-like CD4 T cells were positively and significantly correlated among seroconverted subjects. **(C)** Frequencies of CD45RA+, CCR7^dim^ memory-like CD8 T cells and CD127^dim^ Treg-like CD4 T cells were not correlated among seroconverted and AA– subjects. **(D)** Frequencies of SLEC were not correlated with CD127^dim^ Treg-like CD4 T cells in AA– or seroconverted subjects. **(E)** Frequency of CD45RA+, CCR7^dim^ memory-like CD8 T cells are positively correlated with islet cell antibody (ICA) levels. **(F)** Frequency of SLEC are positively correlated with harmonized insulinoma antigen 2 (IA-2H) levels. Statistical tests fully described in section Materials and Methods.

First, we compared frequencies of T cell subsets using Spearman's Rank-order correlation. We observed no significant correlations among CD127^dim^ Treg-like CD4 T cells with either MAIT cells, SLEC CD8 T cells or CCR7^dim^, CD45RA+ CD8 T cells (Figures 7A,C,D). Alternatively, among seroconverted subjects CD127^bright^ memory CD4 T cells were positively and significantly correlated with the frequency of CD127^dim^ Treg-like CD4 T cells, while there was no significant relationship among AA– subjects (Figure 7B). Further division of the seroconverted group into non-progressor and progressor demonstrated similar strong and significant positive correlations among both groups (data not shown). In total, these correlation data suggest that the frequency dynamics of the CD127^dim^ Treg-like cells do not negatively regulate frequencies of the noted T cell compartments in the periphery. However, they do demonstrate comparable and unique dynamics for CD127^bright^ memory CD4 T cells and CD127^dim^ CD4 T cells among only the seroconverted subjects. This could be due to similar effects driving the reductions observed in these two subsets.

In addition to the correlations comparing frequency of T cell subsets, we also examined relationships between autoantibody levels and frequency of T cells. Such a correlation could reveal if any of these subsets are associated with islet immunity. Interestingly, we observed a positive and significant correlation with islet cell antibody (ICA)values and the CCR7^dim^ CD45RA+, CD8 T cell subset (Figure 7E). Furthermore, we found that the SLEC subset was positively and significantly correlated with harmonized insulinoma antigen 2 (IA-2H) levels (Figure 7F). We observed no significant correlations among other T cell subsets with autoantibody levels (data not shown).

### Seroconverted subjects have significantly elevated ratios of SLEC to both CD127^dim^ Treg-like cells and MAIT cells

**Figure 8:**
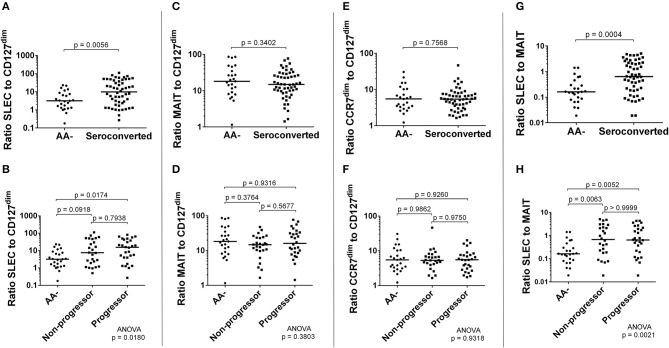
Seroconverted subjects possessed elevated ratios of frequency of SLEC to both frequency of CD127^dim^ Treg-like CD4 cells and frequency of MAIT cells. The ratio of frequency of SLEC to frequency of CD127^dim^ Treg-like cells was significantly elevated among seroconverted subjects **(A)** and extended into both non-progressors and progressors **(B)**. We observed no difference in ratios of either MAIT cells **(C,D)** or CD45RA+, CCR7^dim^ memory-like CD8 T cells **(E,F)** to CD127^dim^ Treg-like CD4 T cells. The ratio of frequency of SLEC to frequency of MAIT cells was significantly elevated among seroconverted subjects **(G)** and extended into both non-progressors and progressors **(H)**. For all figures, bars represent median. Statistical tests fully described in section Materials and Methods.

Along with these correlations, we examined ratios of frequency of effectors to frequency of regulators. Here, MAIT cells were hypothesized to function as either effector or regulator, as a recent report has suggested MAIT cells may have a regulatory function (51). We observed that the ratios of SLEC to CD127^dim^ or MAIT cells were significantly elevated among seroconverted, as well as when divided among non-progressors and progressors (Figures 8A,B,G,H). Alternatively, the ratio of MAIT cells to CD127^dim^ was relatively similar when comparing AA- and seroconverted subjects, including progressors and non-progressors (Figures 8C,D). The ratio of CCR7^dim^ CD45RA+ CD8 T cells to CD127^dim^ CD4 T cells was also similar among AA– and seroconverted subjects, and this continued when seroconverted subjects were divided into non-progressors and progressors (Figures 8E,F).

### SLEC frequencies are strongly associated with CMV IgG levels among seroconverted subjects and CMV+ progressors harbor the highest frequencies of SLEC and associated chronically-activated CD8 T cells

**Figure 9:**
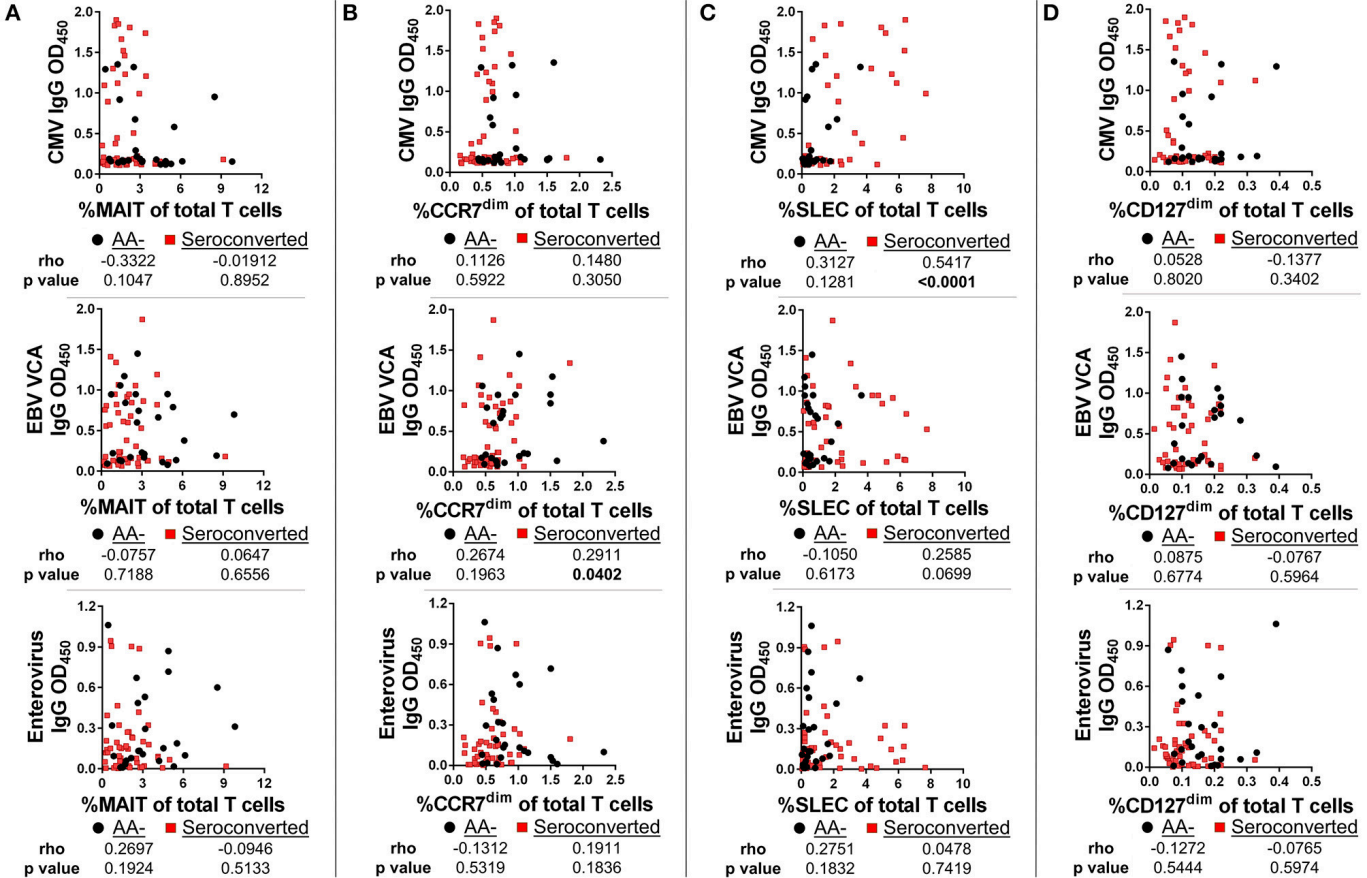
SLEC frequencies are strongly associated with cytomegalovirus IgG levels among seroconverted subjects, but not among controls. We gauged abundance of anti-viral immunoglobulins using ELISA. We then compared frequencies of T cell subsets with IgG abundance. **(A)** Frequency of MAIT cells was not significantly associated with abundance of cytomegalovirus (CMV) IgG, Epstein-Barr Virus viral capsid antigen (EBV VCA) IgG, or enterovirus IgG among seroconverted subjects and controls. **(B)** Frequency of CCR7^dim^, CD45RA+ CD8 T cells was significantly associated with EBV VCA IgG among seroconverted subjects, but not with CMV IgG or enterovirus IgG abundance. **(C)** Frequency of SLEC was significantly associated with CMV IgG abundance among seroconverted subjects but not among controls. We observed no significant relationship among SLEC with either EBV VCA IgG or enterovirus IgG. **(D)** CD127^dim^ Treg-like CD4 T cells were not significantly associated with CMV, EBV VCA, or enterovirus IgG abundance. Statistical tests fully described in section Materials and Methods.

To determine if viral exposure was influencing the T cell changes associated with seroconversion, we tested plasma for abundance of anti-viral immunoglobulins using ELISA. We then performed correlations comparing T cell frequency with relative abundance of viral immunoglobulins as gauged by optical density values. Neither MAIT cells nor CD127^dim^ Treg-like cells were correlated with abundance of CMV, EBV VCA and enterovirus IgG (Figures 9A,D) nor IgM (data not shown). This was not the case for conventional αβ CD8 T cells. We observed a weak but significant association between EBV VCA IgG and CCR7^dim^ CD45RA+ CD8 T cells (Figure 9B). However, there was no significant association among this subset with CMV IgG or enterovirus IgG (Figure 9B), nor any viral IgM tested (data not shown). Finally, we observed a strong and significant association among SLEC and CMV IgG, but not with EBV VCA IgG, enterovirus IgG, nor any viral IgM tested (Figure 9C and data not shown).

**Figure 10:**
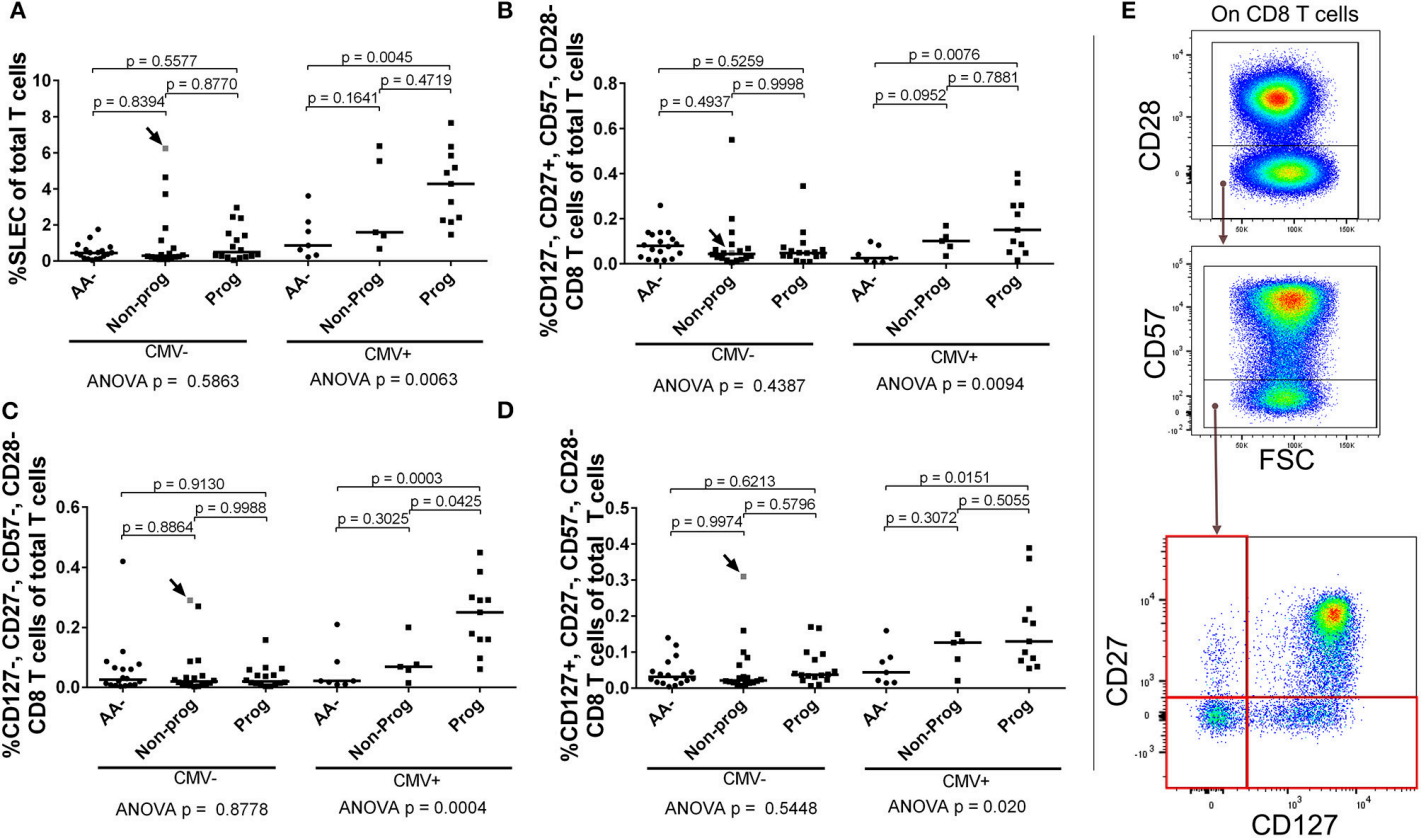
SLEC and associated chronically-stimulated CD8 T cells are significantly elevated among CMV+ progressors. Subjects were stratified as either positive or negative for CMV IgG (CMV+ or CMV–). We then reanalyzed the CD28– CD8 T cell dataset to check for differences associated with CMV among the AA–, non-progressor (Non-prog), and progressor (Prog) groups. **(A)** The SLEC expansion was most prominent among CMV+ progressors. We observed additional chronically-stimulated CD8 T cell subsets that were significantly expanded among the CMV+ progressor group: **(B)** CD127–, CD27+, CD57–, CD28–; **(C)** CD127–, CD27–, CD57–, CD28–; and **(D)** CD127+, CD27–, CD57–, CD28–. The gating for the subsets in **(B–D)** is shown in **(E)**. The CMV–, non-progressor that is colored gray and indicated with an arrow was equivocal for CMV IgG after being tested twice. Bars represent median. Statistical tests fully described in section Materials and Methods.

Since the SLEC subset appeared to be strongly influenced by CMV, we stratified our subjects according to CMV IgG positivity and then retested the entirety of the CD28– CD8 T cell compartment by ANOVA. We observed that the SLEC expansion is greatest among CMV+ progressors (Figure 10A). Furthermore, we observed that the CMV+ progressors possessed elevated frequencies of additional chronically-stimulated CD28– subsets (Figures 10B–E). While these lacked the expression of CD57, they were heterogeneous for CD127 and CD27 expression. In total, these data reveal a relationship among type 1 diabetes progression, terminally-differentiated effector-like CD8 T cells, and existing cytomegalovirus infection among at-risk, seroconverted subjects.

## Discussion

The etiology of type 1 diabetes remains obscure. This is due, in part, to the inaccessibility of the target tissue. Indeed, pancreatic biopsy currently comes with unwanted risk for the donor (52). Analysis of circulating white blood cells as a proxy for solid tissue interrogation allows us to apparently circumvent the problem of pancreas accessibility. Thus, our cross-sectional study was designed to screen multiple T cell subsets from at-risk subjects in order to reveal if immune perturbations are associated with seroconversion and disease progression. To that end, the answer is a clear “yes.” However, this “indirect” analysis is plagued by at least two deficiencies. First, in the absence of a defined causal factor (or set of factors), increases/decreases in frequency of circulating immune cells cannot be explained satisfactorily (53, 54). Second, without follow-up longitudinal analysis of these subjects, such changes in frequency cannot be understood in their dynamic processional roles through time from seroconversion to diagnosis. With these concerns in mind, the following discussion will focus on relevant available reports on comparable immune cell subsets as mere possibilities, as fundamental mechanistic explanations are not available.

Due to their unique function, MAIT cells have been examined in several disease states. Among T1D, the findings are not consistent. We have previously found no change in proportion or number of CD8+ MAIT cells from diabetics in relation to controls (55). Furthermore, we have recently replicated and extended these findings among a second cohort of age-matched juvenile T1Ds and controls (in preparation). Alternatively, Rouxel and colleagues reported reduced frequencies of total MAIT cells to be associated with T1D (51). In the same study, they observed no reduction in proportion of total MAIT cells in their examination of a small cohort of at-risk subjects acquired from TrialNet. Whether these discrepancies result from methodological and/or sampling differences is unclear and independent studies must be conducted for clarification.

Aside from T1D, reductions in circulating MAIT cell populations have been associated with enteric fever (56), several viral infections (57–60), and among other autoimmune diseases and inflammatory disorders such as MS, SLE, RA, and IBD (61–64). Such studies have indicated that MAIT cells are prone to apoptosis in inflammatory milieus, and that reductions in the periphery may co-occur with elevations or reductions in other tissues (57, 62–65). Importantly, type I interferons, perturbed microbiota, viral infection, and increased intestinal permeability have all been associated with the development of T1D (66–69). It remains to be determined if and how these factors impact the MAIT cell lineage and if they are contributing to the deficiencies we have observed.

Along with reduced frequencies of MAIT cells, we observed reductions in several CD4 T cell subsets, which, although phenotypically diverse, all share the expression of CCR4. This is not the first report of CCR4 alterations in T1D. A reduced frequency of CCR4+ CD3+ cells was observed among recently-diagnosed type 1 diabetics (70). Additionally, reduced CCR4 expression following Th1 or Th2 polarization was observed among cord blood T cells from individuals possessing genetic risk alleles for T1D (71). These intriguing results suggest that the CCR4 deficiencies we have observed among seroconverted subjects may have a genetic component and ultimately originate during thymic selection. While our study was neither designed nor powered to explore this question properly, follow-up investigations should be considered highly relevant.

At this point, we cannot define functional roles for the CCR4- expressing subsets we found to be diminished. As described above, there is justification to think that regulatory cells are residing in the CD127^dim^ compartment. Nevertheless, a recent examination of TrialNet samples enquiring into FOXP3 and IL-17-producing CD4 T cells did not report reduced frequencies (72). Due to key methodological differences, it's uncertain how much to extend from this study onto ours. Yet, should we presume that seroconverted subjects are not deficient in regulatory or Th17 cells (both of which are plausible based upon phenotype), we are left with likely Th2 or Th22—type cells. Although relatively unexplored in T1D, Th22-type CD4 T cells have been found in the human gut mucosa (73, 74) and the cytokine IL-22 plays a key role in intestinal defense and integrity (75). Alternatively, the Th2 lineage is generally associated with anti-parasite and allergic responses (76). Interestingly, part of the hygiene hypothesis suggests that the absence of a manageable parasite load is a factor driving increases in the prevalence of autoimmunity (77).

While subset functional status will remain elusive for the time being, the expression of CCR4 clearly indicates responsiveness to MDC, TARC, and CKLF1. Thus, we are currently evaluating plasma from TrialNet subjects for abundance of these and other factors. Importantly, there is some evidence that MDC may play a role in T1D. Elevated MDC transcripts have been found in the duodenal mucosa among T1Ds (78) and MDC can be produced by human islets exposed to a cocktail of inflammatory cytokines (79).

Among conventional CD8 T cells, our screening for T cell aberrations revealed two distinct compartments both associated with islet autoantibody levels. At this point, little can be said regarding the reduced frequency of the CD45RA+, CCR7^dim/−^ CD8 T cell subsets. While their precise characterization indicates central of effector memory-type CD8 T cells, discussions of ontogeny, functionality or specificity of these subsets would be purely speculative and tantamount to hand-waving. Nevertheless, some compelling observations associated with this group do stand out for comment. First, the correlation with ICA levels associates these T cells with beta cell destruction, yet the populations are not expanded among seroconverted subjects and are most reduced among non-progressors. This implies that the “normal” frequencies observed among the progressors could be cryptically pathogenic. Second, the modest association with EBV VCA IgG levels is notable, especially in light of a recent report of a causal role for EBV in autoimmunity (80). Stratification of our subjects according to EBV positivity, as we performed for CMV, did not reveal significant associations with disease progression (data not shown). Therefore, the overall impact of EBV on the development of disease at this point is uncertain.

Fortunately, we can be somewhat more definite in the discussion of the SLEC population, whose phenotype (CD28–, CD57+, CD27–, and CD127–) is indicative of effector status and cytotoxic potential. Furthermore, our data demonstrate that the SLEC subset is strongly associated with CMV IgG levels. Comparable phenotypes have been associated with CMV infection previously (81–87). From our stratifications, we observed that CMV+ progressors possess much higher frequencies of SLEC and related subsets in comparison to CMV+ AA– and non-progressor subjects. This novel observation links a cytotoxic CD8 T cell subset with disease progression and viral exposure. The critical question remaining is what is driving this cellular expansion. An obvious possibility is an acute response to an active CMV infection. To that end, we did not observe CMV IgM positivity among any of the subjects in this study. Nevertheless, we cannot rule out an active viral infection since we were unable to analyze plasma or circulating leukocytes for presence and abundance of viral nucleic acids (88). Such an analysis remains a priority.

In the absence of an active CMV infection, alternate possibilities exist. Following the initial infection, CMV establishes latency and a lifelong residence in the host (89). Over time, reactivation from latency is presumed to drive the expansion of responding CD8 T cells due to chronic antigen exposure (90, 91). In this scenario, the SLEC expansion may represent an exhausted T cell response due to elevated or uncontrolled reactivation of CMV over time. How this could impact beta cells is unknown. Importantly, we observed a positive correlation with IA-2 levels and SLEC frequencies among seroconverted subjects, thereby associating SLEC with beta cell demise and autoimmunity. Whether SLEC could be directly or indirectly responsible for beta cell death remains to be determined. There is *in vitro* evidence that CMV can infect beta cells (92). However, pancreatitis is not normally associated with CMV infection (93), making the direct lysis of CMV-infected beta cells less likely. Aside from direct infection, an answer could be found in the concept of molecular mimicry. It's been reported the CMV major capsid protein shares 64% identity and 73% similarity to the dominant epitope peptide of IA-2 (94). Through that lens, one could imagine the inflated population we have observed to be a polyclonal pool of effectors, some “autoreactive.” Such an expansion could explain the precipitous decline in beta cells that has been observed in the progression to diagnosed diabetes (95), as well as the negative association among CMV-specific CD8 T cells and c-peptide levels among diagnosed type 1 diabetics (96).

In summary, the combined results of our T cell analysis among seroconverted subjects demonstrate reductions of innate-like, memory and regulatory subsets, with a clear expansion of a SLEC compartment (see Supplemental Figure 5 for an interpretation of some of these phenomena). Intriguingly, while the expansion of the SLEC population is most prominent among the progressors, the remaining subsets appear to approach normal mean levels among those same subjects, albeit somewhat bimodally distributed. The causal foundations of these observations are not available. However, they do suggest that measurable and unique immunological changes occur as one moves toward clinical disease. Thus, assuming that seroconversion indicates a triggering event that led to beta cell destruction, all seroconverted subjects share a common history. Concomitantly, seroconverted subjects also have reduced frequencies of MAIT cells, CCR4+ CD4 T cells, and CD45RA+ memory CD8 T cells. For progressors, the advance of beta cell destruction, which is generally foretold by increasing number of autoantibody specificities, is then also foretold by recurrence to relatively normal abundance of these T cell compartments. If, when, and how these populations contribute to autoimmunity remains to be determined. Nevertheless, weaving through this tapestry is the expanded, CMV-associated SLEC compartment, which is clearly present in progressors as well as some non-progressors. This expansion may then be viewed as a hit-and-run or remitting-and-relapsing response to pathogen and/or autoantigen, theories which have been proposed previously (97, 98).

While the paradigm described above could be considered “counterintuitive,” we are wary of talk of intuition in the absence of data. To that end, future efforts should focus on two complementary directions. One, longitudinal analysis of these subjects must be performed to delineate these population dynamics prior to seroconversion, at seroconversion, and closer toward diagnosis. Two, the characterization of the TCR specificity of the SLEC population, elucidation of the functional status of the CCR4-expressing CD4 T cells, and exploration of the fate of the MAIT cell lineage are essential to explicate how the immune system is responding during beta cell demise. In combination, such an approach should reveal which environmental determinants are driving T1D.

## Ethics statement

The human PBMCs and plasma samples we received from TrialNet were deidentified, thus posing no risk to the donor. TrialNet is responsible for consent and IRB approval at their respective institutions. Information on the study can be found here: https://www.trialnet.org/our-research/risk-screening. The University of Nebraska Medical Center Institutional Review Board (IRB) granted approval for purchase of elutriated lymphocytes from the UNMC elutriation core facility.

## Author contributions

RH: experimental design and execution, data and statistical analysis, wrote manuscript. KL-A and KO: experiment execution. VS: data acquisition. LS: statistical analysis. PG: TrialNet liaison. NS: experimental design.

### Conflict of interest statement

The authors declare that the research was conducted in the absence of any commercial or financial relationships that could be construed as a potential conflict of interest.
