# Supplementary figures and images for: Abnormal T Cell Frequencies, Including Cytomegalovirus-Associated Expansions, Distinguish Seroconverted Subjects at Risk for Type 1 Diabetes
Source: Front Immunol. 2018 Oct 22;9:2332. doi: 10.3389/fimmu.2018.02332 (PMC6204396; doi:10.3389/fimmu.2018.02332)

Supplemental Figure 1. Representative gating procedure for all subsets explored

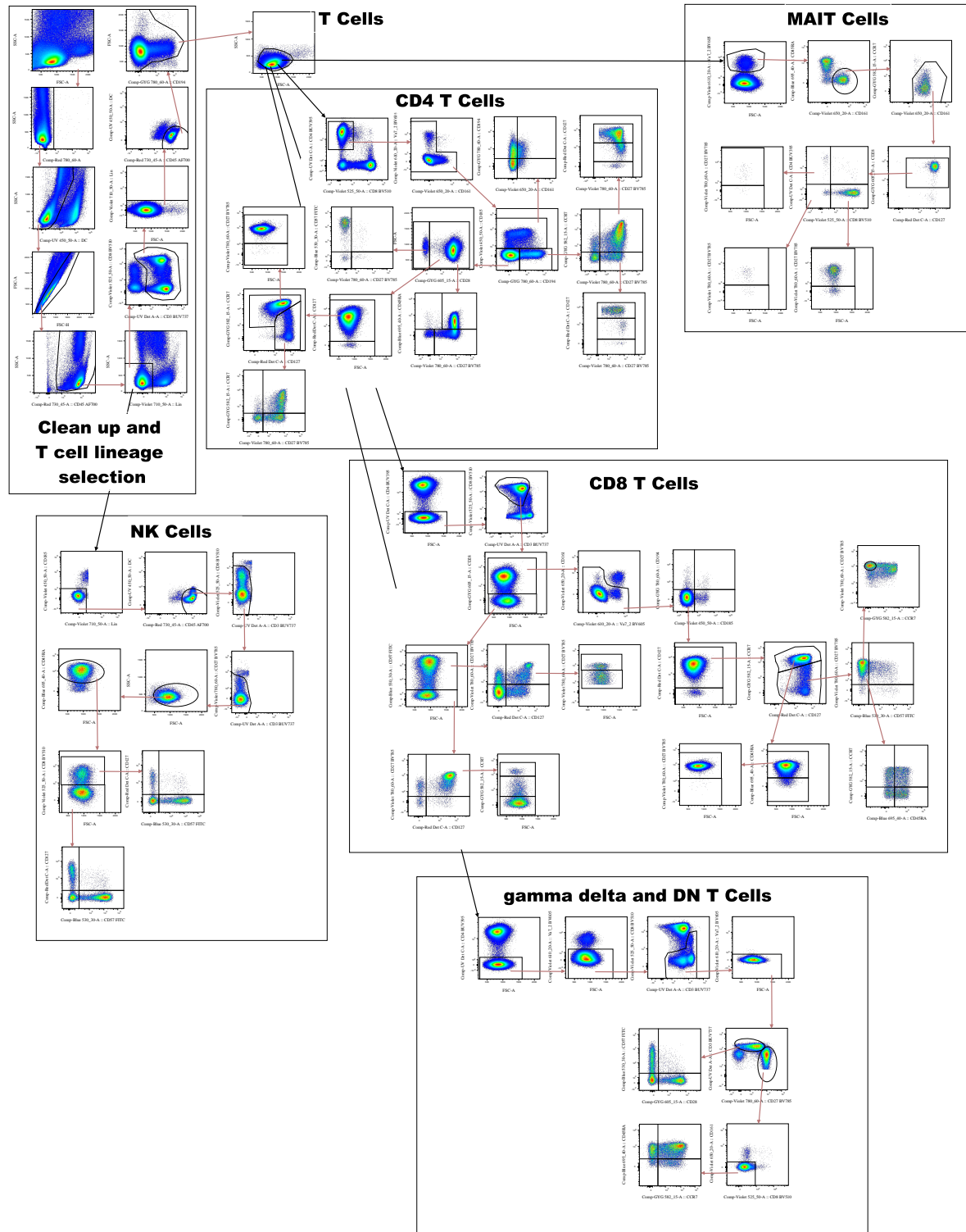

Supplement: Supplementary file 1 [file Data_Sheet_1.pdf]
