# Supplementary material for: Abnormal T Cell Frequencies, Including Cytomegalovirus-Associated Expansions, Distinguish Seroconverted Subjects at Risk for Type 1 Diabetes
Source: Front Immunol. 2018 Oct 22;9:2332. doi: 10.3389/fimmu.2018.02332 (PMC6204396; doi:10.3389/fimmu.2018.02332)
Supplement: Supplementary file 2 [file Data_Sheet_2.pdf]

Supplemental Figure 2

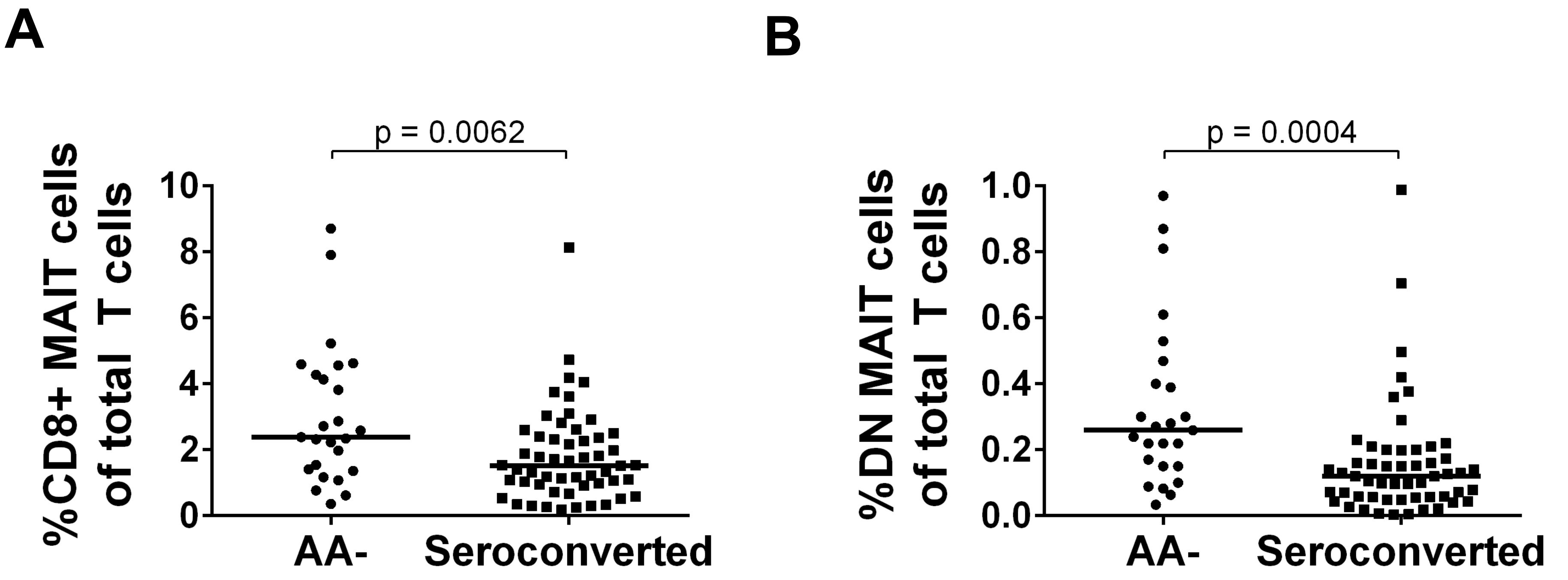

Supplemental Figure 2. MAIT cell reductions were most acute among the CD8+(A) and DN(B)compartments. Gating is shown in figure 1A. For **A** and **B**, bars represent median. Statistical tests fully described in materials and methods.

Supplemental Figure 3. Seroconverted subjects have reduced frequencies of CD45RA+, CCR7- memory-like CD8 T cells.
A. Starting from total CD8 T cells, CD45RA+, CCR7- memory-like CD8 T cells were identified as CD28+, MAIT-, CCR4-, CXCR5-, CD127$^{+/high}$, CCR7-, CD27+, CD45RA+ events. **B.** Seroconverted subjects have reduced frequencies of CD45RA+, CCR7- memory-like CD8 T cells in comparison to AA- subjects. **C.** Upon dividing seroconverted subjects according to disease progression, we observed that reduced frequencies of CD45RA+, CCR7- memory-like CD8 T cells were most prominent among non-progressors. **D.** Descriptive statistics for the frequency of CD45RA+, CCR7- memory-like CD8 T cells of total T cells for all groups compared. For figures **B** and **C**, bars represent median. Statistical tests fully described in materials and methods.

Supplemental Figure 3

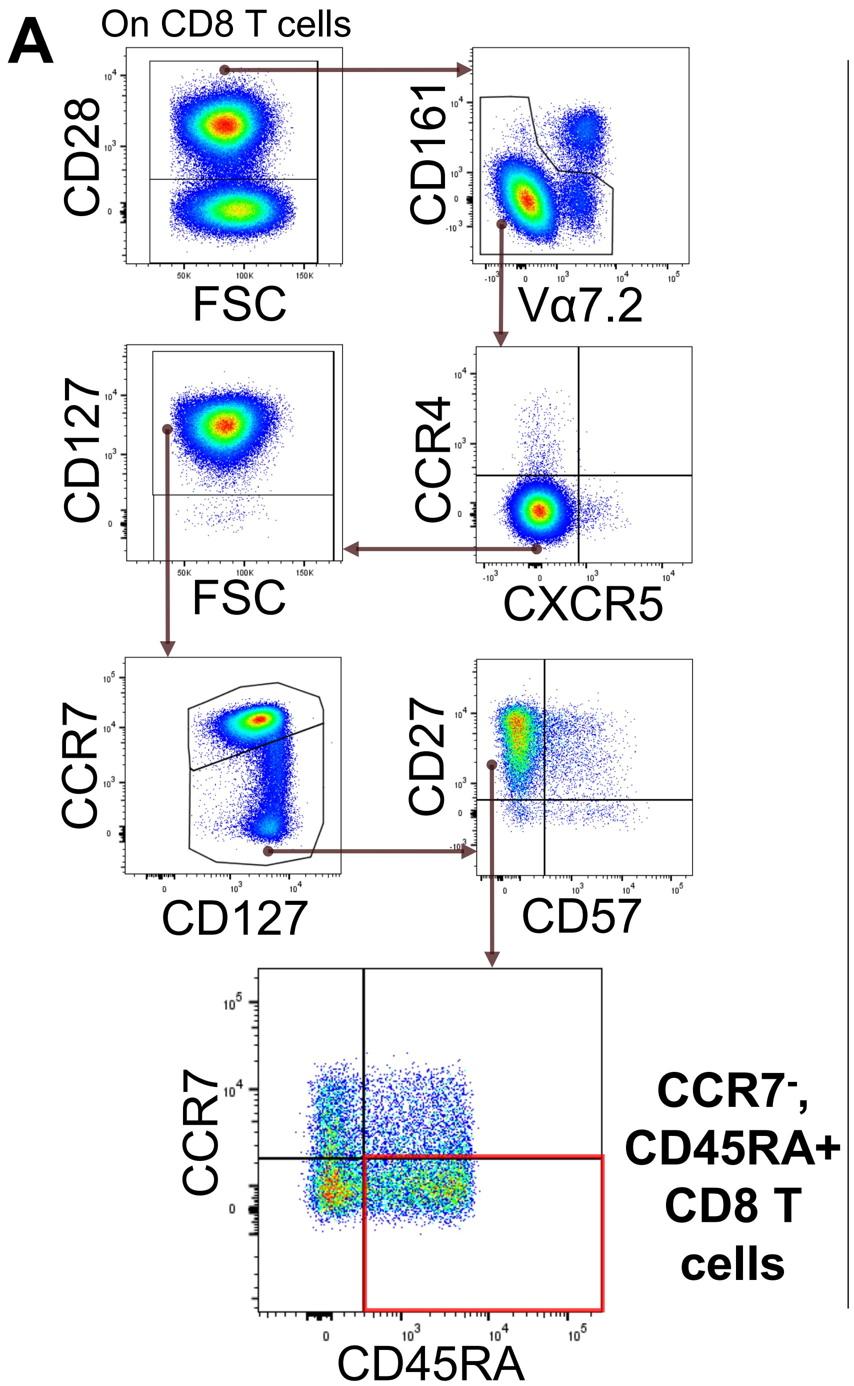

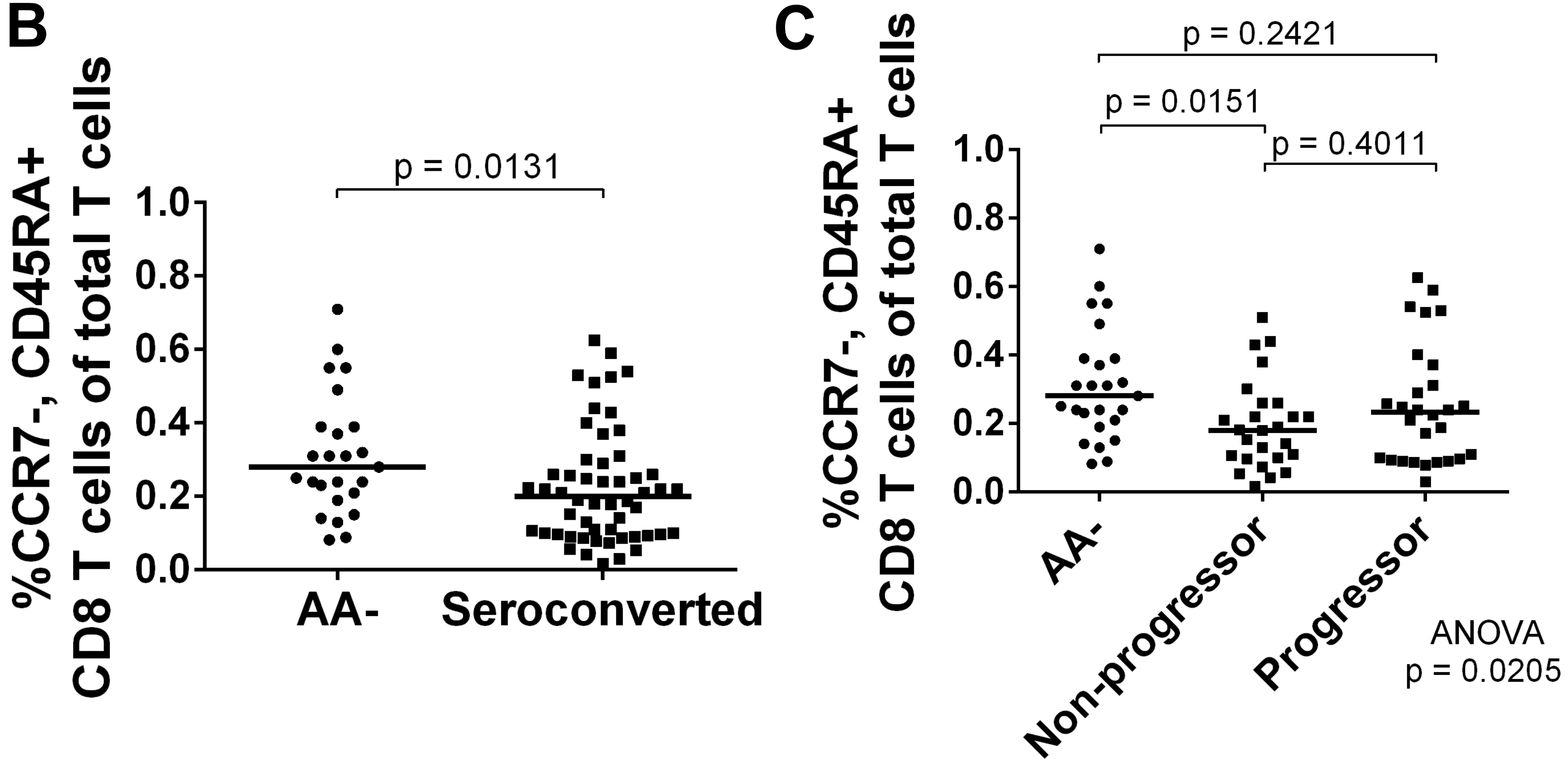

D

%CCR7-, CD45RA+ CD8 T cells of total T cells

	AA-	Seroconverted	Seroconverted: Non-progressor	Seroconverted: Progressor
Mean	0.31	0.22	0.20	0.25
StdDev	0.16	0.16	0.13	0.17
Lower 95% CL for mean	0.24	0.18	0.14	0.18
Upper 95% CL for mean	0.38	0.27	0.25	0.32
Median	0.28	0.20	0.18	0.23
Minimum	0.08	0.02	0.02	0.03
Maximum	0.71	0.63	0.51	0.63

Supplemental Figure 4. Seroconverted subjects have reduced frequencies of T follicular helper-like CD4 T cells. A. Starting from total MAIT- CD4 T cells, T follicular helper-like CD4 T cells were identified as CXCR5+, CCR4+, CD161- events. **B.** Seroconverted subjects have reduced frequencies of T follicular helper-like CD4 T cells in comparison to AA- subjects. **C.** Upon dividing seroconverted subjects according to disease progression, we observed that reduced frequencies of T follicular helper-like CD4 T cells were most prominent among non-progressors. **D.** Descriptive statistics for the frequency of T follicular helper-like CD4 T cells of total T cells for all groups compared. For figures **B** and **C**, bars represent median. Statistical tests fully described in materials and methods.

Supplemental Figure 4

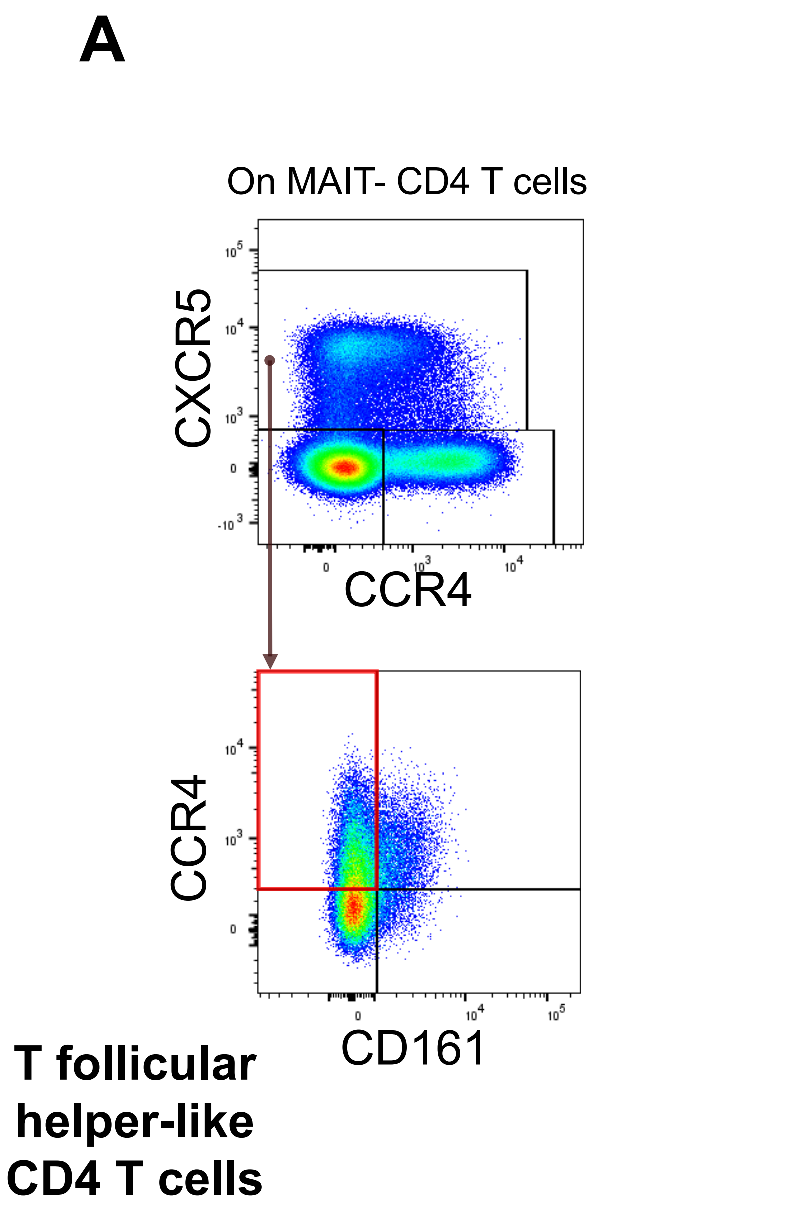

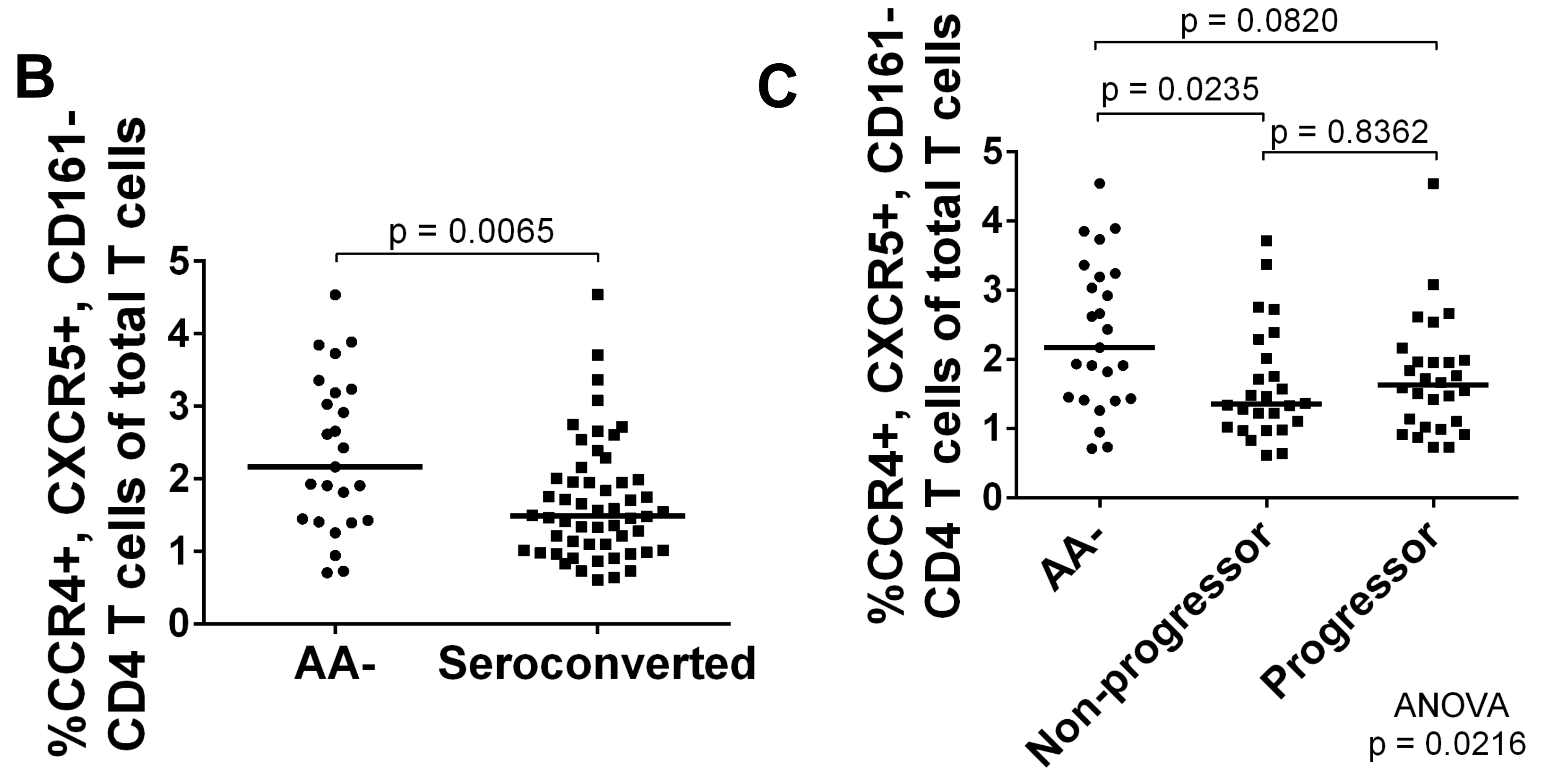

D **%CCR4+, CXCR5+, CD161- CD4 T cells of total T cells**

			Seroconverted	
	AA-	Seroconverted	Non-progressor	Progressor
Mean	2.34	1.68	1.62	1.73
StdDev	1.10	0.81	0.81	0.83
Lower 95% CL for mean	1.90	1.45	1.29	1.41
Upper 95% CL for mean	2.78	1.90	1.95	2.05
Median	2.17	1.50	1.35	1.63
Minimum	0.71	0.61	0.61	0.73
Maximum	4.54	4.54	3.71	4.54

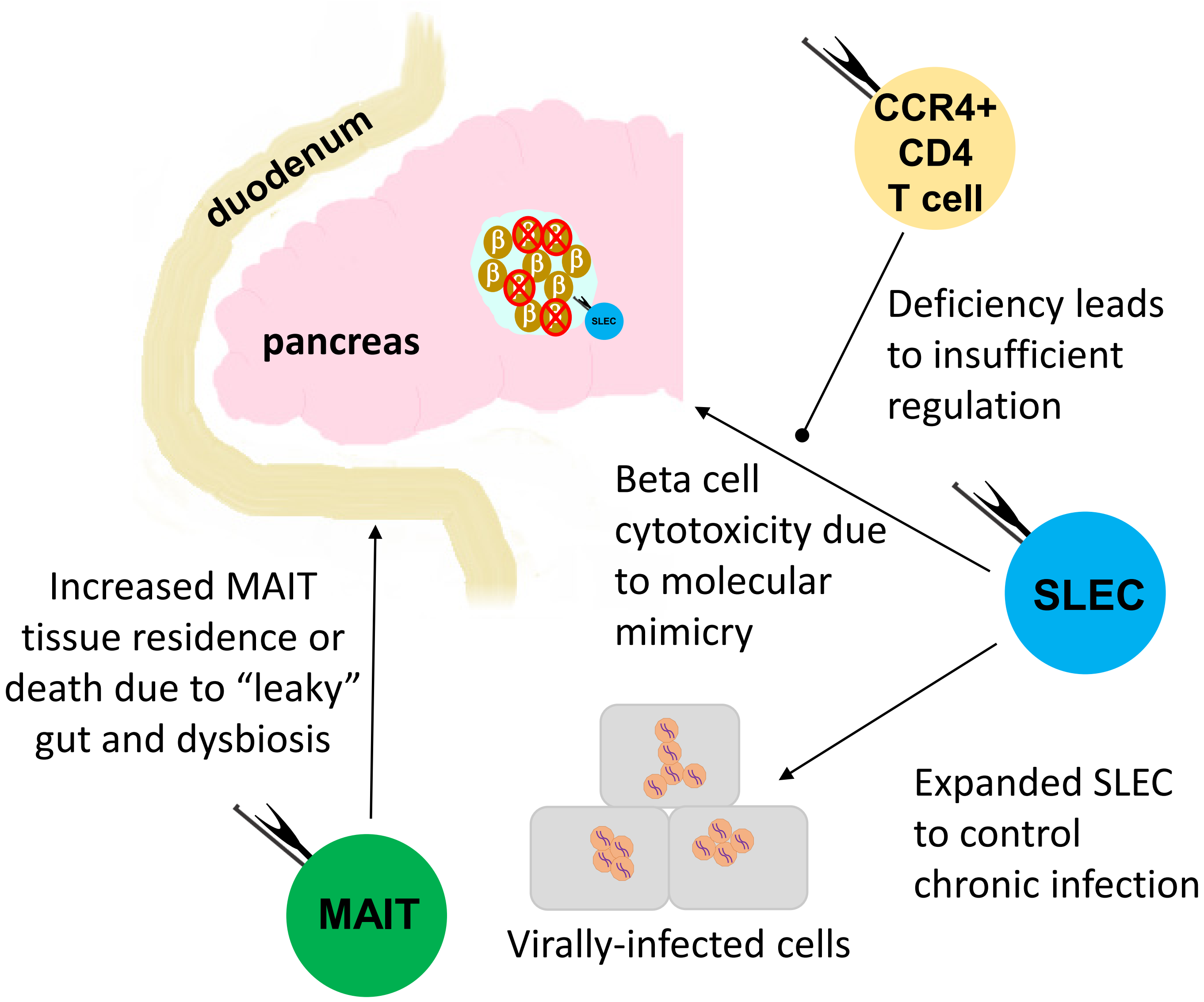

Supplemental Figure 5. An interpretation of some of the T cell alterations described in this report.
